# Besse relaxation difference scheme for a nonlinear integro-differential equation

**DOI:** 10.1371/journal.pone.0327515

**Published:** 2025-08-29

**Authors:** Xinya Peng, Leiwei Li, Jia Zhang

**Affiliations:** 1 College of Computer Science and Mathematics, Central South University of Forestry and Technology, Changsha, Hunan, China; 2 Chenzhou Jinxiang Pharmaceutical Co., Ltd., Chenzhou, Hunan, China; Karlstad University: Karlstads Universitet, SWEDEN

## Abstract

In this paper, Besse relaxation difference and compact difference scheme for a nonlinear integro-differential equation, which is crucial in modeling complex systems with memory and nonlocal effects, are proposed. A Besse relaxation difference scheme is developed by combining Besse relaxation time discretization with second-order spatial discretization, in which the Besse relaxation technique enhanced the accuracy and stability of dealing with nonlinear terms. To further improve spatial accuracy, a fourth-order compact finite difference approximation is used to construct the Besse relaxation compact difference scheme. To verify the effectiveness of the proposed Besse relaxation difference schemes, we have established the unconditional stability and optimal convergence of both methods in the discrete ${\text{L}}^{2}$ norms. Numerical experiments demonstrate that these relaxation schemes attain the predicted convergence rates and high accuracy for smooth solutions, singular solutions, as well as featuring unbounded derivatives solutions.

## 1 Introduction

Efficient and accurate numerical methods are crucial for addressing complex physical and engineering problems, especially when dealing with nonlinear and fractional-order partial differential equations (PDEs) that may exhibit singularities or complicated boundaries. Currently, the mainstream numerical methods primarily include finite difference methods [1], finite element methods [2], and spectral methods [3,4]. These approaches exhibit straightforward structures, are computationally convenient, and have been widely validated for effectively solving common partial differential equations. However, for more complex partial differential equations, these methods generally rely on implicit Newton iteration algorithms [5]. As the mesh becomes more refined, the computational cost escalates sharply, and accuracy may decline, resulting in a substantial increase in overall computational expenses. In contrast, the Besse relaxation difference scheme [6], featuring an explicit-style formulation and superior efficiency, has emerged as a fundamental technique for solving nonlinear PDEs.

A Besse relaxation finite difference and compact difference scheme for the following nonlinear integro-differential equations [7–12] are considered:

$$u_{t}(x,t)+u(x,t)u_{x}(x,t)-I^{\left(\alpha \right)}u_{xx}(x,t)=f(x,t),\;0<x<L,\;0<t\leq T,$$ (1)

where 0<α<1, $I^{\left(\alpha \right)}u_{xx}(x,t)$ is the *α*-order Riemann-Liouville fractional integral:


$$I^{\left(\alpha \right)}u_{xx}(x,t)=\frac{1}{\Gamma (\alpha )}\int_{0}^{t}(t-s{)}^{\alpha -1}u_{xx}(x,s)\;ds,\;t>0.$$

Subject to the boundary conditions

u(0,t)=u(L,t)=0,0≤t≤T, (2)

and the initial condition

$$u(x,0)=u^{0}(x),\;0\leq x\leq L.$$ (3)

Eqs (1)–(3) frequently appear in heat conduction materials with memory effects, population dynamics [10], viscoelasticity problems [11–13], nuclear reaction theory, and others. They are equations that lie between the standard heat conduction equation and the wave equation [7–9].

The Besse relaxation method originates from a linear implicit time-stepping procedure introduced by Besse for discretizing the nonlinear Schrödinger equation [6,14]. Currently, in order to simplify the semi-discretization process of the equation, the second-order Besse relaxation difference scheme has become the mainstream method. Its local well-posedness and convergence have been rigorously proven. Its local well-posedness and convergence have been rigorously established. Notably, Zouraris provided a detailed algorithmic procedure, introduced a combined stability parameter, and derived optimal second-order error estimates [15]. Unlike fully implicit or conventional high-order methods, the second-order Besse relaxation difference scheme does not require extensive iterative computations when handling nonlinear terms, thus effectively simplifying the treatment of these terms. Moreover, it allows flexible spatial discretization without sacrificing temporal accuracy. However, given that the second-order Besse relaxation difference scheme is still limited to second-order spatial accuracy, there is an urgent need for compact variants with higher algebraic complexity to further improve computational accuracy when solving complex partial differential equations [16].

To address this limitation, developing a fourth-order Besse relaxation difference scheme offers an effective way to enhance the solution accuracy. Fourth-order schemes have already been validated in non-relaxation methods for models with highly singular solutions. Representative examples include the work by Zlotnik and Lomonosov [17], in which a non-relaxation approach was restructured into a compact fourth-order difference method on nonuniform grids to improve stability when dealing with strong gradients or unbounded derivatives in acoustic wave equations. Chen and Dai [18] successfully proposed a fourth-order compact absorbing hybrid method for complex boundaries involving absorption or deformation, achieving fourth-order spatial accuracy in KdV equations featuring memory effects. Huang and Yu [19] demonstrated that a high-order compact exponential scheme is capable of effectively handling nonsmooth solutions in time-fractional Black-Scholes models, particularly for financial and wave equations with fractional derivatives. Zhao [20] presented highly accurate compact mixed methods for two-point boundary value problems. These studies have illustrated that reconstructed fourth-order schemes can further enhance numerical accuracy and algorithmic efficiency [21], thereby offering a blueprint to overcome the lack of fourth-order research within Besse relaxation difference schemes.

Inspired by the latest developments in fourth-order schemes for non-relaxation methods, we propose an efficient numerical solver for nonlinear integro-differential equations, achieving the synergistic optimization of spatial and temporal accuracy. Specifically, we introduce two novel space-time discretization schemes : the Besse relaxation difference scheme and the Besse relaxation compact difference scheme, which provide significant advancements under the Besse-type relaxation framework. Among them, the Besse relaxation difference scheme enhances the stability of the solution process, while the Besse compact difference scheme further improves computational accuracy. We then establish the stability and convergence of both schemes, proving that they are unconditionally stable and convergent. Finally, three types of numerical experiments are conducted on equations with singular solutions, smooth solutions, and unbounded derivatives. Example 1, the applicability of our schemes to problems with singular solutions is verified, yielding convergence rates of $O(h^{2}+k^{1+\alpha })$ and $O(h^{4}+k^{1+\alpha })$. A CPU time comparison reveals a 35% to 45% reduction in computational cost relative to the latest non-relaxation Crank–Nicolson method. Example 2 demonstrates that our schemes recover second-order temporal accuracy for smooth solutions and achieve a discrete ${\text{L}}^{2}$-norm estimate, thereby filling a key gap in earlier research. Example 3 evaluates the robustness of the schemes when the regularity of the solution deteriorates, confirming that they still maintain their theoretical order of convergence in this scenario.

The paper is organized as follows: In Sect 2, a Besse relaxation difference scheme is introduced. Sect 3 is devoted to analyze Besse relaxation compact difference scheme. Sects 4 and 5, a rigorous proof of the unconditionally stable nature of the proposed Besse-type relaxation difference schemes are provided. In Sect 6, numerical results that are in perfect agreement with our analysis are shown. Sect 7 provides a brief summary and discussion of the paper.

## 2 Besse relaxation difference scheme

In this section, we will discrete the integral term using the inner product quadrature formula and the time derivative term using the Crank-Nicolson scheme, handle the nonlinear term with the Besse relaxation method, and combine these with the initial and boundary conditions to construct the Besse relaxation difference scheme for Eqs (1)–(3).

Divide the interval [0,*L*] into J+1 equal parts, the interval [0,*T*] into *N* equal parts, and denote $h=L/(J\;+\;1),\;\tau =T/N,\;x_{j}=(j\;+\;1)h,\;0\leq j\leq J\;+\;1,\;t_{n}=n\tau ,\;0\leq n\leq N.$ where *h* is the spatial step size and *τ* is the temporal step size.

For convenience, we introduce some notation. Let the grid functions be defined as:


$$U_{j}^{n}=u(x_{j},t_{n}),\;0\leq j\leq J\;+\;1,\;0\leq n\leq N$$

We introduce the following notation:


$$\Delta U_{j}^{n\;+\;\frac{1}{2}}=\frac{1}{2}\left(U_{j}^{n\;+\;1}\;+\;U_{j}^{n}\right),\;\Delta_{x}U_{j}^{n}=\frac{1}{2h}\left(U_{j\;+\;1}^{n}-U_{j-1}^{n}\right),$$

$$\delta_{t}U_{j}^{n\;+\;\frac{1}{2}}=\frac{1}{\tau }\left(U_{j}^{n\;+\;1}-U_{j}^{n}\right),\;\delta_{x}^{2}U_{j}^{n}=\frac{1}{h^{2}}\left(U_{j-1}^{n}-2U_{j}^{n}\;+\;U_{j\;+\;1}^{n}\right).$$

Next, we consider computing the integral term using the inner product quadrature formula [22].

First, let $t_{n\;+\;\frac{1}{2}}=\left(n\;+\;\frac{1}{2}\right)\tau ,\;0\leq n\leq N-1.$ For $\forall g\in C^{1}([0,T])\cap C^{3}((0,T]),$ as $t\to 0^{+}$, and when $g^{\prime \prime }(t)=O(t^{-\frac{1}{2}})$ and $g^{\prime \prime }(t)=O(t^{-\frac{1}{2}})$, we can numerically approximate the integral term


$$I(g,t):=\int_{0}^{t}(t-s{)}^{\alpha -1}g(s)\;ds,$$

and obtain the coefficients as follows:

$$A_{n}=\frac{1}{\alpha }\left[t_{n}^{\alpha }-\frac{1}{\tau }\int_{t_{n}}^{t_{n\;+\;1}}\theta^{\alpha }d\theta \right],\;c_{0}=\frac{1}{\alpha \tau }\int_{0}^{t_{1}}\theta^{\alpha }d\theta ,$$ (4)

$$c_{p}=\frac{1}{\alpha \tau }\left[\int_{t_{p}}^{t_{p\;+\;1}}\theta^{\alpha }d\theta -\int_{t_{p-1}}^{t_{p}}\theta^{\alpha }d\theta \right],\;p\geq 1.$$ (5)

Thus, we have

$$I(f,t_{n})=A_{n}g(t_{0})\;+\;\sum_{p=0}^{n}c_{p}g(t_{n-p})\;+\;O(\tau^{1\;+\;\alpha }),\;1\leq n\leq N.$$ (6)

From Eqs (4)–(5), we can determine the coefficients of the inner product quadrature formula *A*_*n*_ and *c*_*p*_.

The Besse relaxation method is used primarily to handle nonlinear terms similar to g(u)·u. It first calculates the approximate value of *g*(*u*) at the midpoint $t_{n\;+\;\frac{1}{2}}$ of the time interval $\left[t_{n},t_{n\;+\;1}\right]$, and then computes the approximate value of *u* at the time node $t_{n\;+\;1}$ (see details in [11] ). This method has the advantage of high computational speed when dealing with nonlinear equations. For the nonlinear term *uu*_*x*_ in Eq (1) of this paper, directly discretizing and solving it is very difficult. Our approach is to let g(u)=u and apply the discretization of the first-order central difference directly to *u*_*x*_, thus obtaining the Besse relaxation difference scheme.

The specific algorithm steps are as follows:

**Step 1:** First define $u_{j}^{0}$ as follows: $u_{j}^{0}:=u^{0}\left(x_{j}\right)$, then $u_{j}^{\frac{1}{2}}$ can be obtained from the following equation:

$$\frac{u_{j}^{\frac{1}{2}}-u_{j}^{0}}{\tau /2}\;+\;g(u_{j}^{0})\Delta_{x}\left(\frac{u_{j}^{\frac{1}{2}}\;+\;u_{j}^{0}}{2}\right)-\frac{1}{\Gamma (\alpha )}I\left(u_{xx}(x_{j},\;\cdot \;),t_{0}\right)=\frac{1}{2}\left(f_{j}^{\frac{1}{2}}\;+\;f_{j}^{0}\right),$$ (7)

where $I\left(u_{xx}(x_{j},\;\cdot \;),t_{0}\right)=A_{0}\delta_{x}^{2}u_{j}^{0}\;+\;c_{0}\delta_{x}^{2}u_{j}^{0}.$

**Step 2:** Define $\Phi_{j}^{\frac{1}{2}}$ as follows:


$$\Phi_{j}^{\frac{1}{2}}:=g(u_{j}^{\frac{1}{2}}),$$

then obtain $u_{j}^{1}$ through the following equation:

$$\delta_{t}u_{j}^{\frac{1}{2}}\;+\;\Phi_{j}^{\frac{1}{2}}\Delta_{x}\left(\Delta u_{j}^{\frac{1}{2}}\right)-\frac{1}{\Gamma (\alpha )}I\left(u_{xx}(x_{j},\;\cdot \;),t_{0}\right)=\frac{1}{2}\Delta f_{j}^{\frac{1}{2}},$$ (8)

**Step 3:** Define $\Phi^{n\;+\;\frac{1}{2}}$ as follows:


$$\Phi_{j}^{n\;+\;\frac{1}{2}}:=2g(u_{j}^{n})-\Phi_{j}^{n-\frac{1}{2}},$$

$$I\left(u_{xx}(x_{j},\;\cdot \;),t_{n\;+\;\frac{1}{2}}\right)=\frac{1}{2}(I\left(u_{xx}(x_{j},\;\cdot \;),t_{n}\right)\;+\;I\left(u_{xx}(x_{j},\;\cdot \;),t_{n\;+\;1}\right)).$$

For n≥1, $u_{j}^{n\;+\;\frac{1}{2}}$ can be obtained from the following equation:

$$\delta_{t}u_{j}^{n\;+\;\frac{1}{2}}\;+\;\Phi_{j}^{n\;+\;\frac{1}{2}}\Delta_{x}\left(\Delta u_{j}^{n\;+\;\frac{1}{2}}\right)-\frac{1}{\Gamma (\alpha )}I\left(u_{xx}(x_{j},\;\cdot \;),t_{n\;+\;\frac{1}{2}}\right)=\frac{1}{2}\Delta f_{j}^{n\;+\;\frac{1}{2}},$$ (9)

where


$$I\left(u_{xx}(x_{j},\;\cdot \;),t_{n\;+\;\frac{1}{2}}\right)=\frac{1}{2}\left(A_{n}\delta_{x}^{2}u_{j}^{0}\;+\;\sum_{p=0}^{n}c_{p}\delta_{x}^{2}u_{j}^{n-p}\;+\;A_{n\;+\;1}\delta_{x}^{2}u_{j}^{0}\;+\;\sum_{p=0}^{n\;+\;1}c_{p}\delta_{x}^{2}u_{j}^{n\;+\;1-p}\right).$$

Combined with the initial and boundary conditions:

$$u_{0}^{n}=u_{J\;+\;1}^{n}=0,\;0\leq n\leq N,$$ (10)

and

$$u_{j}^{0}=u^{0}(x_{j}),\;0\leq j\leq J\;+\;1.$$ (11)

Then, we obtain the Besse relaxation difference scheme (7)–(11) for Eqs (1)–(3).

## 3 Besse relaxation compact difference scheme

In this section, to further improve computational accuracy, we will approximate spatial derivatives using the fourth-order compact finite difference method , handle the nonlinear term with the Crank-Nicolson scheme (see details in [12]) and the Besse relaxation method, and combine these with the initial and boundary conditions to construct the Besse relaxation compact difference scheme for Eqs (1)–(3).

For convenience, we introduce some notation. Let the solution vectors on the interior grid points be defined as:


$$\{u^{k}=(u_{1}^{k},u_{2}^{k},\;\cdot \;s,u_{J-1}^{k}{)}^{T}|k=0,\frac{1}{2},1,\;\cdot \;s,n\},$$

where $u_{j}^{k}\approx u(x_{j},t_{n}),j=1,\;\cdot \;s,J-1$ represent indices of discrete points in the interior. Suppose *g* be the defined nonlinear function, and *h* be the spatial grid step size.

Introduce the following notation:


$$g(u^{\frac{1}{2}})=(g(u_{1}^{\frac{1}{2}}),g(u_{2}^{\frac{1}{2}}),\;\cdot \;s,g(u_{J-1}^{\frac{1}{2}}){)}^{T},$$

$$\Delta u^{\frac{1}{2}}=(\frac{u_{j\;+\;1}^{\frac{1}{2}}-u_{j-1}^{\frac{1}{2}}}{2h}{)}_{j=1}^{J-1}.$$

Then, let the right-hand side at the corresponding time levels be defined as:


$$\{f^{\;k}=(f_{1}^{\;\;k},f_{2}^{\;\;k},\;\cdot \;s,f_{J-1}^{\;\;k}{)}^{T}|k=0,\frac{1}{2},\;\cdot \;s,n\}.$$

where $f^{\;k}$ is (J−1)×1 column vector.

To further improve the former scheme fully, we first introduce the fourth-order compact finite difference formulas [23] to approximate the spatial derivatives $U^{\prime }$ and $U^{\prime \prime }$ of Besse relaxation compact difference scheme. We can obtain the values of $U^{\prime }$ and $U^{\prime \prime }$ in the following lemmas 1 and 2.

**Lemma 1.** *([23]) Let*


$$A_{1}={\left[\begin{array}{ccccc} 4 & 1 & 0 & & \\ 1 & 4 & 1 & & \\ & \ddots & \ddots & \ddots & \\ & & 1 & 4 & 1\\ & & 0 & 1 & 4 \end{array}\right]}_{\left(J-1)\times (J-1\right)},$$

*and*


$$H_{1}=\frac{11}{12h}{\left[\begin{array}{c} -u_{0}\\ 0\\ \vdots \\ 0\\ u_{J} \end{array}\right]}_{(J-1)\times 1},\;M_{1}=\frac{3}{h}{\left[\begin{array}{ccccccc} -\frac{4}{3} & 2 & -\frac{4}{9} & \frac{1}{12} & 0 & \;\cdot \;s & 0\\ -1 & 0 & 1 & 0 & 0 & \;\cdot \;s & 0\\ 0 & -1 & 0 & 1 & 0 & \;\cdot \;s & 0\\ & \;\cdot \;s & & \;\cdot \;s & & \;\cdot \;s & \\ 0 & \;\cdot \;s & 0 & -1 & 0 & 1 & 0\\ 0 & \;\cdot \;s & 0 & \frac{-1}{12} & \frac{4}{9} & -2 & \frac{4}{3} \end{array}\right]}_{\left(J-1)\times (J-1\right)}.$$

*Then*


$$U^{\prime }=A_{1}^{-1}\left(M_{1}U\;+\;H_{1}\right),$$

*We have*

$$U^{\prime }(x)=u_{x}(x)\;+\;O(h^{4}).$$ (12)

**Lemma 2.** *([23]) Let*


$$A_{2}={\left[\begin{array}{ccccccc} 14 & -5 & 4 & -1 & 0 & \;\cdot \;s & 0\\ 1 & 10 & 1 & 0 & 0 & \;\cdot \;s & 0\\ 0 & 1 & 10 & 1 & 0 & \;\cdot \;s & 0\\ & \;\cdot \;s & & \;\cdot \;s & & \;\cdot \;s & \\ 0 & \;\cdot \;s & 0 & 0 & 1 & 10 & 1\\ 0 & \;\cdot \;s & 0 & -1 & 4 & -5 & 14 \end{array}\right]}_{\left(J-1)\times (J-1\right)},$$

*and*


$$H_{2}=\frac{12}{h^{2}}{\left[\begin{array}{c} u_{0}\\ 0\\ \vdots \\ 0\\ u_{J} \end{array}\right]}_{(J-1)\times 1},\;M_{2}=\frac{12}{h^{2}}{\left[\begin{array}{ccccc} -2 & 1 & 0 & & \\ 1 & -2 & 1 & & \\ & \ddots & \ddots & \ddots & \\ & & 1 & -2 & 1\\ & & 0 & 1 & -2 \end{array}\right]}_{\left(J-1)\times (J-1\right)}.$$

*Then*


$$U^{\prime \prime }=A_{2}^{-1}\left(M_{2}U\;+\;H_{2}\right).$$

*We have*

$$U^{\prime \prime }(x)=u_{xx}(x)\;+\;O(h^{4}).$$ (13)

Based on Eqs (12)–(13), we now derive the Besse relaxed compact finite difference scheme. A detailed explanation is provided as follows:

**Step 1:** First define $u_{j}^{0}$ as follows: $u_{j}^{0}:=u^{0}\left(x_{j}\right)$, then $u^{\frac{1}{2}}$ can be obtained from the following equation:

$$\frac{u^{\frac{1}{2}}-u^{0}}{\tau /2}\;+\;G^{\frac{1}{2}}A_{1}^{-1}\left(M_{1}\Delta u^{\frac{1}{2}}\;+\;H_{1}\right)-\frac{1}{\Gamma (\alpha )}A_{2}^{-1}\left(M_{2}u^{0}\;+\;H_{2}\right)=\frac{f^{\frac{1}{2}}\;+\;f^{0}}{2}.$$ (14)

Initially, we define $v^{\frac{1}{2}}$ as $A_{1}^{-1}\left(M_{1}\Delta u^{\frac{1}{2}}\;+\;H_{1}\right)$, where $v^{\frac{1}{2}}$ is a (J−1)×1 column vector. Then, perform an element-wise operation with $g(u^{\frac{1}{2}})$. To unify matrix operations, we define:


$$G^{\frac{1}{2}}=\text{diag}(g(u_{1}^{\frac{1}{2}}),g(u_{2}^{\frac{1}{2}}),\;\cdot \;s,g(u_{J-1}^{\frac{1}{2}})),$$

Consequently, the term becomes $G^{\frac{1}{2}}v^{\frac{1}{2}}.$

**Step 2:** Define auxiliary variables $\Phi^{\frac{1}{2}}$:


$$\Phi^{\frac{1}{2}}=g\left(u^{\frac{1}{2}}\right),$$

and correspondingly, we have:


$$\Phi^{\frac{1}{2}}:=\text{diag}(\Phi_{1}^{\frac{1}{2}},\Phi_{2}^{\frac{1}{2}},\;\cdot \;s,\Phi_{J-1}^{\frac{1}{2}})=\text{diag}(g(u_{1}^{\frac{1}{2}}),g(u_{2}^{\frac{1}{2}}),\;\cdot \;s,g(u_{J-1}^{\frac{1}{2}})),$$

then, using this auxiliary variable, we solve for $u^{1}$:

$$\frac{u^{1}-u^{\frac{1}{2}}}{\tau /2}\;+\;\Phi^{\frac{1}{2}}A_{1}^{-1}(M_{1}\Delta u^{\frac{1}{2}}\;+\;H_{1})-\frac{1}{\Gamma (\alpha )}A_{2}^{-1}(M_{2}u^{0}\;+\;H_{2})=\frac{f^{\frac{1}{2}}\;+\;f^{1}}{2}.$$ (15)

**Step 3:** For n≥1, we first define:


$$\Phi_{j}^{n\;+\;\frac{1}{2}}:=2g(u_{j}^{n})-\Phi_{j}^{n-\frac{1}{2}},$$

in vector form:


$$\Phi^{n\;+\;\frac{1}{2}}:=2g(u^{n})-\Phi^{n-\frac{1}{2}},$$

simultaneously, we construct the diagonal matrix:


$$\Phi^{n\;+\;\frac{1}{2}}=\text{diag}(\Phi_{1}^{n\;+\;\frac{1}{2}},\Phi_{2}^{n\;+\;\frac{1}{2}},\;\cdot \;s,\Phi_{J-1}^{n\;+\;\frac{1}{2}}),$$

then, we solve for $u^{n\;+\;1}$:

$$\frac{u^{n\;+\;1}-u^{n}}{\tau }\;+\;\Phi^{n\;+\;\frac{1}{2}}A_{1}^{-1}(M_{1}\Delta u^{n\;+\;\frac{1}{2}}\;+\;H_{1})\;\;-\frac{1}{\Gamma (\alpha )}A_{2}^{-1}(M_{2}I(u_{xx}(\;\cdot \;),t_{n\;+\;\frac{1}{2}})\;+\;H_{2})=\frac{f^{n\;+\;\frac{1}{2}}}{2},$$ (16)

where $I(u_{xx}(\;\cdot \;),t_{n\;+\;\frac{1}{2}})$ is defined as:


$$I(u_{xx}(\;\cdot \;),t_{n\;+\;\frac{1}{2}})=\frac{1}{2}(A_{0}A_{2}^{-1}(M_{2}U^{0}\;+\;H_{2})\;+\;\sum_{p=0}^{n}c_{p0}A_{2}^{-1}(M_{2}U^{n-p}\;+\;H_{2})\;\;+\;A_{n\;+\;1}A_{2}^{-1}(M_{2}U^{0}\;+\;H_{2})\;+\;\sum_{p=0}^{n\;+\;1}c_{p0}A_{2}^{-1}(M_{2}U^{n\;+\;1-p}\;+\;H_{2})),$$

I(·) vectorially denotes the discretization applied to each grid point, yielding a vector of the same dimension.

The initial and boundary conditions for the problem are defined as follows:

$$u_{0}^{n}=u_{J\;+\;1}^{n}=0,\;0\leq n\leq N,$$ (17)

and

$$u_{j}^{0}=u^{0}(x_{j}),\;0\leq j\leq J\;+\;1.$$ (18)

Then, we obtain the Besse relaxation compact difference scheme (14)–(18) for Eqs (1)–(3).

## 4 Stability analysis

### 4.1 The stability of Besse relaxation difference scheme

Consider a linearized version of the original problem (ignoring the source term *f*). Let $u(x,t)=\bar{u}\;+\;\tilde{u}(x,t)$, where $\bar{u}$ is a constant. We replace the nonlinear term $u\;u_{x}$ by $\bar{u}\;{\tilde{u}}_{x}$. Our goal is to examine the stability of the resulting numerical scheme for the perturbation $\tilde{u}$.

**Definition 4.1** (Two-Step Besse Relaxation Scheme). *Denote the discrete solution by ${\tilde{u}}_{j}^{n}\approx \tilde{u}(x_{j},t_{n})$ for grid points $x_{j}=j\;h$ and time levels $t_{n}=n\;\tau$. We write one full time step $\left(t_{n}\to t_{n\;+\;1}\right)$ as two half-steps:*


$$t_{n}\;\to \;t_{n\;+\;\frac{1}{2}}\;\to \;t_{n\;+\;1}.$$

*For simplicity of notation, consider the step from *t**_*0*_ *to *t**_*1*_:

*(i)* $t_{0}\to t_{\frac{1}{2}}$:
  $$\frac{{\tilde{u}}_{j}^{\frac{1}{2}}-{\tilde{u}}_{j}^{0}}{\tau /2}\;+\;\;\bar{u}\;\partial_{x}\;(\frac{{\tilde{u}}_{j}^{\frac{1}{2}}\;+\;{\tilde{u}}_{j}^{0}}{2})\;-\;\frac{A_{0}}{\Gamma (\alpha )}\;\partial_{xx}{\tilde{u}}_{j}^{0}\;=\;0,$$ (19)
*(ii)* $t_{\frac{1}{2}}\to t_{1}$:
  $$\frac{{\tilde{u}}_{j}^{1}-{\tilde{u}}_{j}^{\frac{1}{2}}}{\tau /2}\;+\;\;\bar{u}\;\partial_{x}\;(\frac{{\tilde{u}}_{j}^{1}\;+\;{\tilde{u}}_{j}^{\frac{1}{2}}}{2})\;-\;\frac{A_{0}}{\Gamma (\alpha )}\;\partial_{xx}{\tilde{u}}_{j}^{0}\;=\;0.$$ (20)

**Lemma 3** (Stability of (i)). *Under the linearized setting of Definition 4.1, let ${\tilde{U}}_{j}^{m}$ be approximated by a Fourier mode $e^{\;i\;\kappa \;j\;h}$. Substituting into (19) yields an amplification factor*


$$\xi_{\frac{1}{2}}\;=\;\frac{\;1-\frac{\tau }{2}\;(S-i\;C_{0}\;D)\;}{\;1\;+\;\frac{\tau }{2}\;(S-i\;C_{0}\;D)\;},\;C_{0}\;=\;\frac{A_{0}}{\Gamma (\alpha )\;D}.$$

*Let $\lambda =\frac{\tau \;C_{0}\;D}{2}\geq 0$ and s=sin(κh/2). Then*


$$|\xi_{\frac{1}{2}}{|}^{2}=\frac{\;1-2\;\lambda \;s^{2}\;+\;\lambda \;s^{4}\;}{\;1\;+\;2\;\lambda \;s^{2}\;+\;\lambda \;s^{4}\;}\leq 1,$$

*which implies $|\xi_{\frac{1}{2}}|\leq 1$.*

*Proof:* The result follows from direct substitution of ${\tilde{u}}_{j}^{m}\sim e^{\;i\;\kappa \;j\;h}$ into (19) and taking the modulus of the resulting amplification factor. Since $s^{2}\geq 0$, the numerator does not exceed the denominator, which concludes $|\xi_{\frac{1}{2}}|\leq 1$. □

**Lemma 4** (Stability of (ii)). *For the second half-step (20), an analogous Fourier argument shows that if*


$$\xi_{1}\;=\;\frac{\;1-\frac{\tau }{2}\;(S-i\;C_{0}\;D)\;}{\;1\;+\;\frac{\tau }{2}\;(S-i\;C_{0}\;D)\;},$$

*then $|\xi_{1}|\leq 1$ also holds.*

*Proof:* This follows exactly the same steps as in Lemma 3, but substituting into (20) and noting that the spatial difference operator $\partial_{xx}$ again contributes a non-positive real part to the exponent. Hence the resulting amplification factor has modulus not exceeding 1. □

**Theorem 4.1** (Unconditional stability of the two-step scheme). *Combining Lemmas 3 and 4, one full time step of size *τ* satisfies*


$$|\xi |\;=\;|\xi_{1}\circ \xi_{\frac{1}{2}}|\;\leq \;1,$$

*ensuring unconditional stability of the linearized scheme described in Definition 4.1.*

*Proof:* A single time step from *t*_0_ to *t*_1_ is implemented by two half-steps. By Lemma 3, the amplification factor for the first half-step satisfies $|\xi_{\frac{1}{2}}|\leq 1$, and similarly by Lemma 4 for the second half-step we get $|\xi_{1}|\leq 1$. The product of two factors whose moduli do not exceed 1 continues to satisfy |ξ|≤1. Hence the scheme remains stable for all choices of *τ* and *h*, i.e. it is unconditionally stable. □

Consider a more general Crank-Nicolson Besse relaxation scheme

$$\delta_{t}u_{j}^{n\;+\;\frac{1}{2}}\;\;+\;\;\bar{u}\;\delta_{x}\;(\Delta_{x}u_{j}^{n\;+\;\frac{1}{2}})\;-\;\frac{1}{\Gamma (\alpha )}\;{ℐ}_{t_{n\;+\;\frac{1}{2}}}^{\left(\alpha \right)}[\partial_{xx}u_{j}{]}^{n\;+\;\frac{1}{2}}\;=\;0,$$ (21)

where $u_{j}^{n}=\xi^{n}e^{\;i\;\kappa \;j\;h}$.

**Theorem 4.2** (Stability of the generalized Crank-Nicolson Besse relaxation scheme). *Under the same Fourier mode assumption, the amplification factor associated with (21) is*


$$\xi \;=\;\frac{1\;-\;\frac{\tau }{2}\;[S(\kappa )\;-\;C_{n}(\xi )\;D(\kappa )]}{1\;\;+\;\;\frac{\tau }{2}\;[S(\kappa )\;-\;C_{n}(\xi )\;D(\kappa )]},$$

*where S(κ) is purely imaginary and $C_{n}(\xi )\;D(\kappa )$ has a nonnegative real part. Consequently, |ξ|≤1, ensuring unconditional stability for the generalized scheme.*

*Proof:* By inspection of the discrete operator in (21), the real part contributed by $C_{n}(\xi )\;D(\kappa )$ is nonnegative, while S(κ) is purely imaginary (due to the symmetric spatial discretization and the form of the half-step integration). Therefore, the denominator and numerator in the fraction for ξ form a conjugate-like pair in such a way that |ξ|≤1. The details mirror those of Lemma 1 and Lemma 2, extended to a time-fractional or Crank–Nicolson framework. □

**Remark 1.** *These results establish the unconditional stability of both the two-step Besse relaxation scheme (Theorem 4.2) and its generalization (Theorem 4.3). Extensions to fully nonlinear problems typically require a linearization or fixed-point iteration at each step, but the core stability argument remains similar once the spatial operator is recognized to have a non-positive real part in its Fourier transform.*

### 4.2 The stability of Besse relaxation compact difference scheme

Let $u^{n}=(u_{j}^{n}{)}_{j=1}^{J-1}\in {\mathbb{R}}^{J-1}$ be the numerical solution vector of interior grid points at time *t*_*n*_. Define two discrete operators:


$$D^{\left(1\right)}\;=\;A_{1}^{-1}(M_{1}\;+\;H_{1}),\;D^{\left(2\right)}\;=\;A_{2}^{-1}(M_{2}\;+\;H_{2}),$$

where *A*_*k*_ are symmetric positive-definite (SPD) matrices, *M*_*k*_ are skew-symmetric matrices, and *H*_*k*_ incorporate Dirichlet boundary conditions. Below, $\langle u,v\rangle_{h}=h\;\sum_{j=1}^{J-1}u_{j}v_{j}$ denotes the discrete inner product.

**Lemma 5.** *If *A**_*1*_ *is SPD and *M**_*1*_ *is skew-symmetric, then*


$$D^{\left(1\right)}\;=\;A_{1}^{-1}(M_{1}\;+\;H_{1})$$

*satisfies*


$$(D^{\left(1\right)}{)}^{T}\;=\;-\;D^{\left(1\right)},$$

*and*


$$\langle D^{\left(1\right)}u,\;v\rangle_{h}\;=\;-\;\langle u,\;D^{\left(1\right)}v\rangle_{h}\;\forall \;u,v.$$

*Hence D*^*(1)*^ *acts like a discrete divergence/gradient operator that is antisymmetric under this inner product.*

**Lemma 6.** *If *A**_*2*_ *is SPD and *M**_*2*_ *is skew-symmetric, then*


$$D^{\left(2\right)}\;=\;A_{2}^{-1}(M_{2}\;+\;H_{2})$$

*is negative semi-definite, i.e.*


$$\langle D^{\left(2\right)}\;u,\;u\rangle_{h}\;\leq \;0\;\text{for all discrete vectors }u.$$

We now analyze the linearized form of the Besse relaxation compact difference scheme. In order to perform an energy estimate, consider the linearized form of the equation for $\tilde{u}$:

$$\frac{{\tilde{u}}^{\;n\;+\;1}-{\tilde{u}}^{\;n}}{\tau }\;\;+\;\;\frac{\bar{u}}{2}\;D^{\left(1\right)}\;({\tilde{u}}^{\;n\;+\;1}\;+\;{\tilde{u}}^{\;n})\;-\;\frac{1}{\Gamma (\alpha )}\;D^{\left(2\right)}\;(I_{n\;+\;\frac{1}{2}})\;=\;0,$$ (22)

where


$$I_{n\;+\;\frac{1}{2}}\;=\;\frac{1}{2}[I_{t_{n}}^{\left(\alpha \right)}({\tilde{u}}_{xx})\;\;+\;\;I_{t_{n\;+\;1}}^{\left(\alpha \right)}({\tilde{u}}_{xx})]\;=\;\frac{1}{2}\sum_{p=0}^{\;n\;+\;1}\omega_{n\;+\;1-p}\;{\tilde{u}}^{\;p},$$

and $\omega_{k}>0$ come from the quadrature coefficients $A_{n},\;c_{n}$ (cf. Eq. (6)).

**Theorem 4.1** (Unconditional stability). The linearized Besse relaxation compact difference scheme (22) is unconditionally stable in the discrete ${\text{L}}^{2}$ norm. Specifically, for all n≥0,


$$\|{\tilde{u}}^{n\;+\;1}{\|}_{h}^{2}\;\leq \;\|{\tilde{u}}^{n}{\|}_{h}^{2}.$$

*Proof:* To derive a discrete energy estimate, we multiply (22) by $\left({\tilde{U}}^{n\;+\;1}\;+\;{\tilde{U}}^{n}\right)$ in the discrete inner product:

$$\langle \frac{{\tilde{u}}^{n\;+\;1}-{\tilde{u}}^{n}}{\tau },\;{\tilde{u}}^{n\;+\;1}\;+\;{\tilde{u}}^{n}\rangle_{h}\;\;+\;\;\bar{u}\;\langle D^{\left(1\right)}({\tilde{u}}^{\;n\;+\;\frac{1}{2}}),\;{\tilde{u}}^{n\;+\;1}\;+\;{\tilde{u}}^{n}\rangle_{h}\;-\;\frac{1}{\Gamma (\alpha )}\langle D^{\left(2\right)}(I_{n\;+\;\frac{1}{2}}),\;{\tilde{u}}^{n\;+\;1}\;+\;{\tilde{u}}^{n}\rangle_{h}\;=\;0.$$ (23)

#### Time difference term.

Using the identity


$$\langle {\tilde{u}}^{n\;+\;1}-{\tilde{u}}^{n},\;{\tilde{u}}^{n\;+\;1}\;+\;{\tilde{u}}^{n}\rangle_{h}=\|{\tilde{u}}^{n\;+\;1}{\|}_{h}^{2}-\|{\tilde{u}}^{n}{\|}_{h}^{2},$$

the first term in (23) becomes


$$\langle \frac{{\tilde{u}}^{n\;+\;1}-{\tilde{u}}^{n}}{\tau },\;{\tilde{u}}^{n\;+\;1}\;+\;{\tilde{u}}^{n}\rangle_{h}=\frac{1}{\tau }\;[\|{\tilde{u}}^{n\;+\;1}{\|}_{h}^{2}-\|{\tilde{u}}^{n}{\|}_{h}^{2}].$$

#### Discrete nonlinear term.

By Lemma 5, the operator ${\text{D}}^{\left(1\right)}$ is antisymmetric under $\langle \;\cdot \;,\;\cdot \;\rangle_{h}$, so


$$\langle D^{\left(1\right)}({\tilde{u}}^{\;n\;+\;\frac{1}{2}}),\;{\tilde{u}}^{n\;+\;1}\;+\;{\tilde{u}}^{n}\rangle_{h}=0,$$

which implies no energy contribution from the transport part.

#### Numerical integral term.

By Lemma 6, ${\text{D}}^{\left(2\right)}$ is negative semi-definite, hence


$$\langle D^{\left(2\right)}(I_{n\;+\;\frac{1}{2}}),\;{\tilde{u}}^{n\;+\;1}\;+\;{\tilde{u}}^{n}\rangle_{h}\;\leq \;0.$$

Putting these results into (23) yields:

$$\frac{\|{\tilde{u}}^{n\;+\;1}{\|}_{h}^{2}-\|{\tilde{u}}^{n}{\|}_{h}^{2}}{\tau }\;-\;\frac{1}{\Gamma (\alpha )}\langle D^{\left(2\right)}(I_{n\;+\;\frac{1}{2}}),\;{\tilde{u}}^{n\;+\;1}\;+\;{\tilde{u}}^{n}\rangle_{h}\;=\;0,$$ (24)

and the last term is non-positive. Therefore,


$$\|{\tilde{u}}^{n\;+\;1}{\|}_{h}^{2}\;\leq \;\|{\tilde{u}}^{n}{\|}_{h}^{2},\;\forall \;n\geq 0.$$

This completes the proof of the unconditional stability in the discrete ${\text{L}}^{2}$ norm. □

## 5 Convergence analysis

### 5.1 The convergence of Besse relaxation difference scheme

**Theorem 5.1** (Local truncation error). *With source terms and nonlinear effects included, each step of the Besse relaxation difference scheme has a local truncation error (LTE) on the order of*


$${LTE}_{m}\;=\;O(\tau^{1\;+\;\alpha }\;\;+\;\;h^{2}),$$

*depending on whether the solution is smooth or has singular memory effects (i.e., *α*-fractional).*

**Remark 2.** *When the fractional parameter *α* is involved, the time stepping may exhibit reduced convergence order in the temporal part (on the order of $\tau^{1\;+\;\alpha }$) if the solution or memory kernel introduces additional singularities. In contrast, for sufficiently smooth solutions without strong fractional singularities, the scheme often behaves as a standard second-order method in time, hence $\tau^{2}$.*

**Theorem 5.2** (Global error estimate). *Let ${\text{u}}^{\text{n}}$ be the exact solution and ${\text{U}}^{\text{n}}$ be the numerical solution from the Besse relaxation difference scheme. Define the global error $e^{n}=U^{n}\;-\;u^{n}$. Under the stability condition (|ξ|≤1) and assuming the local truncation error in Theorem 5.1, we have*


$$\|e^{n}\|\;\leq \;\|e^{0}\|\;\;+\;\;\sum_{m=0}^{n-1}\tau \;\|{LTE}_{m}\|\;=\;O(\tau^{1\;+\;\alpha }\;+\;h^{2}).$$

*Hence the second-order Besse-relaxed scheme achieves $O(\tau^{2}\;+\;h^{2})$ order for smooth solutions, or $O(\tau^{1\;+\;\alpha }\;+\;h^{2})$ if the fractional parameter *α* dominates.*

*Proof:* We begin by noting that each time step of the scheme contributes a local truncation error ${LTE}_{m}=O(\tau^{1\;+\;\alpha }\;+\;h^{2})$, as stated in Theorem 5.1. By a standard Lax-Richtmyer argument for linear (or linearized) stable schemes, the global error satisfies


$$\|e^{n}\|\;\leq \;\|e^{0}\|\;\;+\;\;\sum_{m=0}^{n-1}\tau \;\|{LTE}_{m}\|.$$

Since each ${LTE}_{m}=O(\tau^{1\;+\;\alpha }\;+\;h^{2})$, straightforward summation over *m* = 0 to *n*–1 gives


$$\|e^{n}\|\;=\;O(\tau^{1\;+\;\alpha }\;+\;h^{2}).$$

In the case of sufficiently smooth solutions (where fractional singularities do not degrade the time accuracy), the temporal order effectively becomes $O(\tau^{2})$, yielding a second-order scheme in time and space. This completes the proof. □

### 5.2 The convergence of Besse relaxation compact difference scheme

**Theorem 5.1** (Local truncation error). *For each time step of the Besse relaxation compact difference scheme, let ${LTE}_{n}$ denote the local truncation error (including Besse-type weights and fractional operator discretization). Then, under suitable regularity assumptions on the exact solution ${\text{u}}^{\text{n}}$, one has*


$${LTE}_{n}\;=\;O(\tau^{1\;+\;\alpha }\;\;+\;\;h^{4}).$$

**Remark 3.** *If the fractional exponent *α* introduces strong memory effects, the temporal error term is typically $O(\tau^{1\;+\;\alpha })$. For smoother cases or small *α*, one may approach $O(\tau^{2})$ in time. In any case, the fourth-order compact approach raises the spatial accuracy to $\text{O}({\text{h}}^{4})$, improving upon standard second-order schemes.*

**Theorem 5.2** (Global error estimate). *Let ${\text{U}}^{\text{n}}$ be the numerical solution produced by the fourth-order compact Besse-relaxed scheme, and let ${\text{u}}^{\text{n}}$ be the exact solution. Denote the global error by $e^{n}=U^{n}\;-\;u^{n}$. Then, repeating the energy estimate and using the local truncation error from Theorem 5.1, we obtain*


$$\|e^{n\;+\;1}{\|}_{h}^{2}\;\leq \;\|e^{n}{\|}_{h}^{2}\;\;+\;\;C\;\tau \;\|{LTE}_{n}{\|}_{h}^{2}\;=\;O(\tau^{1\;+\;\alpha }\;+\;h^{4}),$$

*which implies*


$$\|e^{n}{\|}_{h}\;=\;O\;(\tau^{\frac{1\;+\;\alpha }{2}}\;+\;h^{2}).$$

*Hence this scheme achieves higher-order accuracy in both time and space.*

*Proof:* Let the error is $e^{n}=U^{n}-u^{n}$. By construction, ${\text{e}}^{\text{n}}$ satisfies the same discrete relation as in (22), except for an additional inhomogeneous term ${LTE}_{n}$ on the right-hand side. Applying the discrete energy estimate in the same manner as (23)–(24), we get


$$\|e^{n\;+\;1}{\|}_{h}^{2}\;\leq \;\|e^{n}{\|}_{h}^{2}\;\;+\;\;C\;\tau \;\|{LTE}_{n}{\|}_{h}^{2}.$$

From Theorem 5.1, each ${LTE}_{n}=O(\tau^{1\;+\;\alpha }\;+\;h^{4})$, so


$$\|e^{n\;+\;1}{\|}_{h}^{2}\;\leq \;\|e^{n}{\|}_{h}^{2}\;\;+\;\;C^{\prime }\;\tau \;(\tau^{1\;+\;\alpha }\;+\;h^{4}).$$

A straightforward telescoping or summation argument over *n* then shows


$$\|e^{n}{\|}_{h}^{2}\;=\;O(\tau^{1\;+\;\alpha }\;+\;h^{4}),$$

thus, there is


$$\|e^{n}{\|}_{h}\;=\;O\;(\tau^{\frac{1\;+\;\alpha }{2}}\;+\;h^{2}).$$

Therefore, the method attains fourth-order accuracy in space and up to second-order in time or $\tau^{(1\;+\;\alpha )/2}$ if dominated by fractional effects. This completes the proof. □

## 6 Numerical experiments

In this section, we present several numerical examples to illustrate the effectiveness and accuracy of the Besse relaxation difference scheme (7)–(11) and the compact difference scheme (14)–(18). To compare their performance, we evaluate errors and convergence rates under various spatial and temporal refinements, focusing on both point-wise and overall measures of solution accuracy.

We define two types of errors relative to the exact solution $\left\{u_{j}^{n}\right\}$:


$$E_{\infty }(J,N)\;=\;\underset{1\;\leq n\;\leq N}{\underset{1\;\leq j\;\leq J}{\max}}|\;U_{j}^{n}-u_{j}^{n}|,\;E_{2}(J,N)\;=\;\sqrt{\;h\;\tau \;\sum_{n=1}^{N}\sum_{j=1}^{J}(U_{j}^{n}-u_{j}^{n}{)}^{2}}.$$

where h=L/(J+1) is the spatial mesh size and τ=T/N is the time step. The former ($E_{\infty }$) tracks the maximum point-wise error over all grid nodes and time levels, while the latter (*E*_2_) represents the root-mean-square discrepancy across the entire spatiotemporal grid.

To quantify how the error decreases as *J* and *N* grow, we use the following indicators:


$${\text{Rate}}^{x}\;=\;\log_{2}\;(\frac{E_{\infty }(2J,N)}{E_{\infty }(J,N)}),\;{\text{Rate}}^{t}\;=\;\log_{2}\;(\frac{E_{\infty }(J,2N)}{E_{\infty }(J,N)}),$$

$${\text{Rate}}_{x}^{\left(2\right)}\;=\;\log_{2}\;(\frac{E_{2}(2J,N)}{E_{2}(J,N)}),\;{\text{Rate}}_{t}^{\left(2\right)}\;=\;\log_{2}\;(\frac{E_{2}(J,2N)}{E_{2}(J,N)}),$$

where $\log_{2}(\;\cdot \;)$ estimates the order of accuracy by comparing the error when doubling the number of spatial or temporal steps. These definitions are consistent with those in (8)–(18) and enable a direct comparison of the $\;L^{\infty }$-norm and the $\;L^{2}$-norm.

In the subsequent examples, we will report both $E_{\infty }$ and *E*_2_ under various mesh sizes (*J*) and time steps (*N*), along with their associated convergence rates. This allows us to assess the numerical behavior of the two proposed schemes, especially in terms of stability and achievable accuracy for different solution types. Unless stated otherwise, *J* = 1024 and *N* = 4096 are used as baseline values.

**Example 1:** Assuming the exact solution of (1)–(3) is


$$u(x,t)=\sin\pi x-\frac{t^{\alpha \;+\;1}}{\Gamma (\alpha \;+\;2)}\sin2\pi x.$$

The initial condition is u(x,0)=sinπx, and the right-hand side term is:


$$f(x,t)=\left[\sin\pi x-\frac{t^{\alpha \;+\;1}}{\Gamma (\alpha \;+\;2)}\sin2\pi x\right]\left[\pi \cos\pi x-\frac{2\pi t^{\alpha \;+\;1}}{\Gamma (\alpha \;+\;2)}\cos2\pi x\right]$$

$$-\frac{t^{\alpha }\sin2\pi x}{\Gamma (\alpha \;+\;1)}-\frac{1}{\Gamma (\alpha )}\left[\frac{-\pi^{2}\sin\pi x}{\alpha }\;t^{\alpha }\;+\;\frac{4\pi^{2}\sin2\pi x}{\Gamma (2\alpha \;+\;2)}\;t^{\alpha \;+\;1}\Gamma (\alpha )\right].$$

For the case in which *u*_*tt*_(*x*,*t*) exhibits a singularity at *t* = 0, we compare the Besse relaxation difference scheme and the Besse relaxation compact difference scheme by examining both the maximum-norm and discrete ${\text{L}}^{2}$-norm errors.

**Fig 1:**
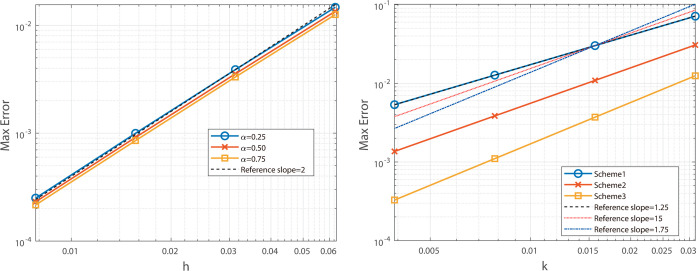
Illustrates the spatial convergence order (left) and temporal convergence order (right) of the Besse relaxation difference scheme at α=0.25, 0.50, and 0.75.

**Fig 2:**
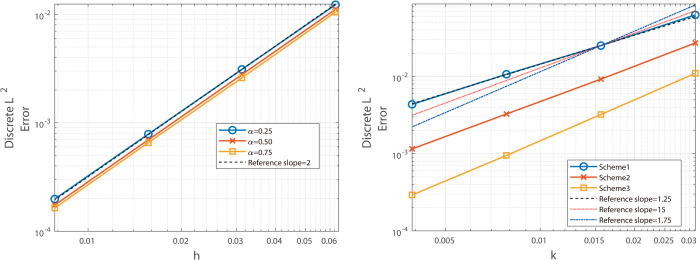
Illustrates the spatial convergence order (left) and temporal convergence order (right) of the Besse relaxation difference scheme at α = 0.25, 0.50, and 0.75.

To validate the spatial accuracy of the second-order scheme, we fix *N* = 4096 and vary *J*, reporting maximum-norm results for α=0.25,0.50,0.75 in Table 1, alongside corresponding ${\text{L}}^{2}$-norm errors in Table 3. From these data, the scheme achieves second-order spatial convergence and a temporal rate close to 1+α. Fig 1 illustrates that the slopes of the maximum-norm error curves align with theoretical expectations, while Fig 2 confirms a similar trend for the ${\text{L}}^{2}$-norm.

**Table 1:** Maximum errors and convergence orders with varying step sizes of the Besse Relaxation Difference Scheme (temporal grid number N=4096 and spatial grid number J=1024 fixed).

α	Spatial convergence order				Time convergence order			
	*J*	$E_{\infty }(h,\tau )$	${Rate}^{x}$	CPU(s)	*N*	$E_{\infty }(h,\tau )$	${Rate}^{t}$	CPU(s)
0.25	16	1.4697e-2	*	1.8286	32	7.1411e-2	*	2.2123
	32	3.8847e-3	1.9196	2.1051	64	3.0138e-2	1.2446	4.5729
	64	9.9756e-4	1.9613	5.5631	128	1.2705e-2	1.2462	9.4028
	128	2.5018e-4	1.9954	12.7636	256	5.3489e-3	1.2481	18.2695
0.50	16	1.3478e-2	*	1.9036	32	3.0721e-2	*	2.4839
	32	3.5510e-3	1.9243	1.9314	64	1.0882e-2	1.4973	4.3301
	64	9.1457e-4	1.9570	5.5885	128	3.8509e-3	1.4983	9.1678
	128	2.3122e-4	1.9838	13.0534	256	1.3621e-3	1.4994	18.2089
0.75	16	1.2575e-2	*	1.5953	32	1.2467e-2	*	2.2142
	32	3.3185e-3	1.9220	2.3458	64	3.7069e-3	1.7499	4.3942
	64	8.5333e-4	1.9594	5.6509	128	1.1025e-3	1.7495	8.9153
	128	2.1629e-4	1.9802	13.4627	256	3.2782e-4	1.7498	18.1666

**Table 2:** ${\text{L}}^{2}$ errors and convergence orders with varying step sizes of the Besse Relaxation Difference Scheme (temporal grid number N=4096 and spatial grid number J=1024 fixed).

α	Spatial convergence order				Time convergence order			
	*J*	$E_{2}(h,\tau )$	Rate${\;}_{x}^{\left(2\right)}$	CPU(s)	*N*	$E_{2}(h,\tau )$	Rate${\;}_{t}^{\left(2\right)}$	CPU(s)
0.25	16	1.2247e-2	*	3.0049	32	6.2873e-2	*	4.6987
	32	3.1057e-3	1.9742	5.0013	64	2.5167e-2	1.2423	8.9855
	64	7.8621e-4	1.9820	8.2513	128	1.0708e-2	1.2485	14.4606
	128	1.9813e-4	1.9890	9.5106	256	4.3580e-3	1.2493	33.5423
0.50	16	1.1123e-2	*	1.8945	32	2.7207e-2	*	4.2862
	32	2.7820e-3	1.9964	4.2312	64	9.2458e-3	1.4968	9.1254
	64	6.9631e-4	1.9983	7.7035	128	3.2683e-3	1.4979	18.5481
	128	1.7433e-4	1.9981	9.5414	256	1.1544e-3	1.4990	37.3611
0.75	16	1.0458e-2	*	1.1565	32	1.0996e-2	*	3.4515
	32	2.5940e-3	1.9796	2.5688	64	2.2198e-3	1.7458	10.0195
	64	6.5491e-4	1.9835	4.7173	128	9.4925e-4	1.7473	16.4823
	128	1.6375e-4	1.9849	9.4720	256	2.9172e-4	1.7495	29.3727

**Table 3:** Maximum errors and convergence orders with varying step sizes of the Besse Relaxation Compact Difference Scheme (temporal grid number N=4096 and spatial grid number J=1024 fixed).

α	Spatial convergence order				Time convergence order			
	*J*	$E_{\infty }(h,\tau )$	${\text{Rate}}^{\text{x}}$	CPU(s)	*N*	$E_{\infty }(h,\tau )$	${\text{Rate}}^{\text{t}}$	CPU(s)
0.25	16	8.8679e-3	*	10.5005	32	1.4178e-1	*	2.4483
	32	6.4725e-4	3.9365	12.2225	64	5.7173e-2	1.2437	5.6630
	64	3.9019e-5	3.9074	22.8864	128	2.3790e-2	1.2458	15.2265
	128	2.6827e-6	3.9937	63.6645	256	8.9867e-3	1.2489	17.3352
0.50	16	8.0739e-3	*	10.5968	32	2.0238e-2	*	2.4839
	32	5.5438e-4	3.9439	11.2566	64	6.8623e-3	1.4928	4.3301
	64	3.5528e-5	3.9240	24.8879	128	2.4859e-3	1.4947	9.1678
	128	2.3948e-6	3.9438	36.0921	256	8.0983e-4	1.4991	18.2089
0.75	16	7.2415e-3	*	15.3389	32	8.9867e-3	*	13.2507
	32	4.8743e-4	3.9897	25.5537	64	2.3810e-3	1.7475	38.4733
	64	2.9974e-5	3.9965	65.1153	128	7.0726e-4	1.7488	64.3375
	128	2.1576e-6	3.9939	98.1240	256	2.3760e-4	1.7494	78.5537

**Fig 3:**
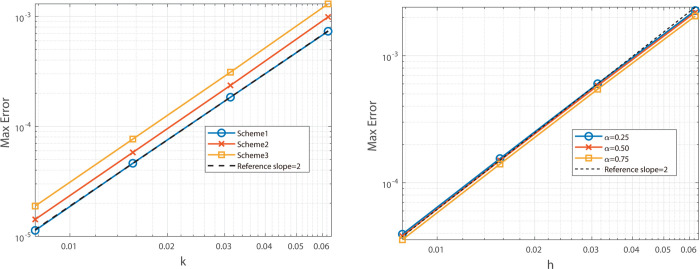
Illustrates the temporal convergence order (left) and spatial convergence order (right) of the Besse relaxation difference scheme at α = 0.25, 0.50, and 0.75.

**Fig 4:**
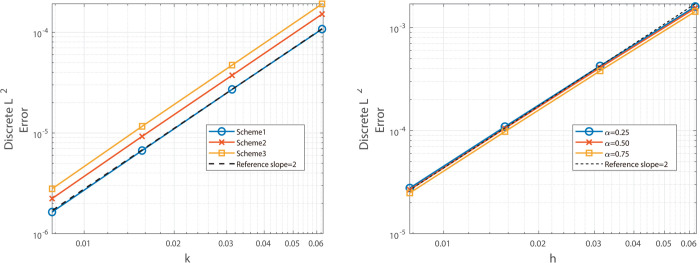
Illustrates the temporal convergence order (left) and spatial convergence order (right) of the Besse relaxation difference scheme at α = 0.25, 0.50, and 0.75.

Similarly, under the same grid settings, the fourth-order Besse relaxation compact scheme attains fourth-order spatial accuracy and a temporal order near 1+α, as evidenced by the maximum-norm results in Table 3 and the ${\text{L}}^{2}$-norm data in Table 4. This consistency is visually reinforced by Figs 3 and 4, where the error slopes match the predicted rates. These comparisons highlight the clear advantage of the compact approach in delivering higher spatial precision.

**Table 4:** ${\text{L}}^{2}$ errors and convergence orders with varying step sizes of the Besse Relaxation Compact Difference Scheme (temporal grid number N=4096 and spatial grid number J=1024 fixed).

α	Spatial convergence order				Time convergence order			
	*J*	$E_{2}(h,\tau )$	Rate${\;}_{x}^{\left(2\right)}$	CPU(s)	*N*	$E_{2}(h,\tau )$	Rate${\;}_{t}^{\left(2\right)}$	CPU(s)
0.25	16	9.7789e-4	*	15.2859	32	1.3598e-2	*	6.6382
	32	6.2011e-5	3.9824	27.7653	64	5.7173e-3	1.2458	14.7034
	64	3.9079e-6	3.9907	31.6538	128	2.3790e-3	1.2466	20.3718
	128	2.4088e-7	3.9982	45.9277	256	1.0250e-3	1.2483	22.5638
0.50	16	8.0418e-4	*	21.1878	32	2.0258e-3	*	3.7287
	32	5.1028e-5	3.9714	19.7357	64	7.5510e-4	1.4945	9.3906
	64	3.2037e-6	3.9944	56.5537	128	2.8859e-4	1.4976	17.2667
	128	2.0013e-7	3.9980	24.4799	256	1.0183e-4	1.4991	25.4824
0.75	16	7.0347e-4	*	41.1666	32	7.7354e-4	*	13.7724
	32	4.5076e-5	3.9594	50.4728	64	2.0770e-4	1.7455	29.0614
	64	2.8061e-6	3.9642	66.6870	128	6.9821e-5	1.7470	41.0010
	128	1.7529e-7	3.9826	105.3017	256	2.0632e-5	1.7489	70.5891

These results highlight the clear advantage of the fourth-order compact scheme in achieving higher spatial accuracy compared to the second-order scheme.Nevertheless, due to the weak singularity at *t* = 0, both methods remain limited by the fractional parameter *α* in time accuracy. Future work could explore alternative temporal strategies or relaxation refinements to mitigate this restriction and further improve performance for singular initial-value problems.

To further demonstrate the computational efficiency of our proposed Besse relaxation methods, we compare them against a *classical Crank–Nicolson scheme with Picard iteration* for the nonlinear algebraic equations. Specifically, we apply the Crank–Nicolson time discretization and at each time step $t_{n\;+\;1}$, we solve


$$\frac{U_{j}^{n\;+\;1}-U_{j}^{n}}{\tau }\;\;+\;\;\frac{1}{2}(U_{j}^{n\;+\;1}\;+\;U_{j}^{n})\;\delta_{x}\;(\frac{1}{2}(U^{n\;+\;1}\;+\;U^{n}){)}_{j}\;-\;\frac{1}{\Gamma (\alpha )}[\frac{1}{2}(I_{n\;+\;1,j}^{\left(\alpha \right)}\;+\;\;I_{n,j}^{\left(\alpha \right)})]\;=\;f_{j}^{\left(n\;+\;\frac{1}{2}\right)},$$

via the iterative update


$$U_{j}^{n\;+\;1,(m\;+\;1)}\;=\;\text{PicardIteration}(U_{j}^{n\;+\;1,(m)},\;U_{j}^{n},\;I_{n,j}^{\left(\alpha \right)},\;f_{j}^{\left(n\;+\;\frac{1}{2}\right)}),$$

until


$$\|\;U^{n\;+\;1,(m\;+\;1)}\;-\;U^{n\;+\;1,(m)}{\|}_{\infty }\;<\;10^{-10}.$$

All spatial differences ($\delta_{x},\;\delta_{xx}$) and the fractional history terms $\left\{I_{n,j}^{\left(\alpha \right)}\right\}$ are defined consistently with our Besse relaxation scheme (see (7)–(11) and (14)–(18) for details). By running **Example 1** under identical mesh and time-step settings for both the non-relaxation Picard method and our relaxation approach, we obtain a direct CPU-time comparison that highlights how the Besse relaxation scheme can substantially reduce computational cost while preserving numerical accuracy, as shown in Tables 5 and 6.

**Table 5:** CPU time comparison for different methods of the Besse Relaxation Difference Scheme (temporal grid number *N* = 4096 and spatial grid number *J* = 1024 fixed).

*α*	Besse Relaxation Difference Scheme				Crank–Nicolson scheme			
	*J*	${\text{CPU}}_{x}(s)$	*N*	${\text{CPU}}_{t}(s)$	*J*	${\text{CPU}}_{x}(s)$	*N*	${\text{CPU}}_{t}(s)$
0.25	16	1.8286	32	2.2123	16	3.3247	32	4.0224
	32	2.1051	64	4.5729	32	3.8275	64	8.3144
	64	5.5631	128	9.4028	64	10.1147	128	17.0960
	128	12.7636	256	18.2695	128	23.2065	256	33.2173
0.50	16	1.9036	32	2.4839	16	3.4611	32	4.5162
	32	1.9314	64	4.3301	32	3.5116	64	7.8729
	64	5.5885	128	9.1678	64	10.1609	128	16.6687
	128	13.0534	256	18.2089	128	23.7335	256	33.1071
0.75	16	1.5953	32	2.2142	16	2.9005	32	4.0258
	32	2.3458	64	4.3942	32	4.2651	64	7.9894
	64	5.6509	128	8.9153	64	11.2744	128	16.2996
	128	13.4627	256	18.1666	128	25.4776	256	32.9902

**Table 6:** CPU time comparison for different methods of the Besse Relaxation Compact Difference Scheme (temporal grid number *N* = 4096 and spatial grid number *J* = 1024 fixed).

α	Besse Relaxation Compact Difference Scheme				Crank–Nicolson scheme			
	*J*	${\text{CPU}}_{x}(s)$	*N*	${\text{CPU}}_{t}(s)$	*J*	${\text{CPU}}_{x}(s)$	*N*	${\text{CPU}}_{t}(s)$
0.25	16	10.5005	32	2.4483	16	16.0546	32	3.8666
	32	12.2225	64	5.6630	32	17.9946	64	8.7123
	64	22.8864	128	15.2265	64	34.2098	128	23.4254
	128	63.6645	256	17.3352	128	98.9454	256	23.6695
0.50	16	10.5968	32	2.4839	16	16.3028	32	3.8214
	32	11.2566	64	4.3301	32	17.3186	64	6.6617
	64	24.8879	128	9.1678	64	37.2889	128	15.0243
	128	36.0921	256	18.2089	128	56.5267	256	27.6695
0.75	16	15.3389	32	13.2507	16	24.5980	32	21.3852
	32	25.5537	64	38.4733	32	37.3134	64	54.9897
	64	65.1153	128	64.3375	64	98.8774	128	99.9808
	128	98.1240	256	78.5537	128	148.9860	256	123.1518

**Example 2:** Assuming the exact solution of (1)–(3) is


$$u(x,t)=t^{2}sin\pi x,$$

The initial condition is u(x,0)=0, and the right-hand side term is:


$$f=2t\sin\pi x-\frac{\pi }{2}t^{4}\sin2\pi x\;+\;\frac{\pi^{2}sin\pi x}{\Gamma (\alpha \;+\;3)}t^{2\;+\;\alpha }\Gamma (3).$$

**Fig 5:**
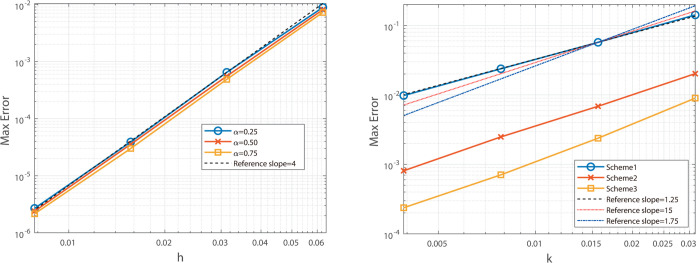
Illustrates the spatial convergence order (left) and temporal convergence order (right) of Besse 358 relaxation compact difference scheme at α=0.25, 0.50, and 0.75.

**Fig 6:**
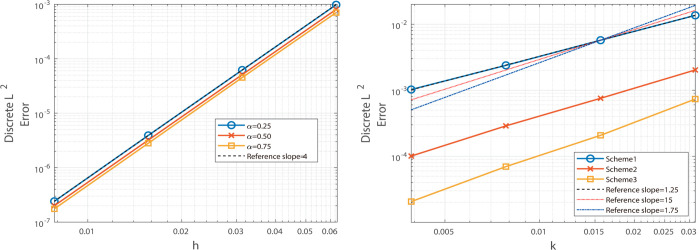
Illustrates the spatial convergence order (left) and temporal convergence order (right) of Besse relaxation compact difference scheme at α = 0.25, 0.50, and 0.75.

**Fig 7:**
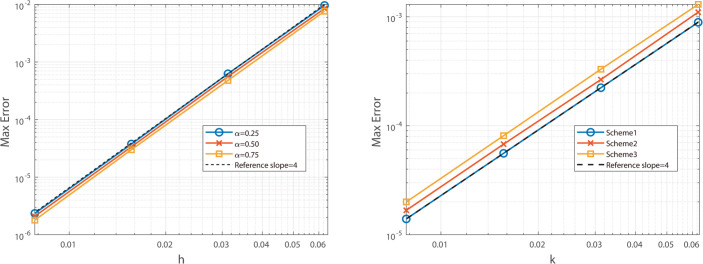
Illustrates the temporal convergence order (left) and spatial convergence order (right) of Besse relaxation compact difference scheme at α = 0.25, 0.50, and 0.75.

**Fig 8:**
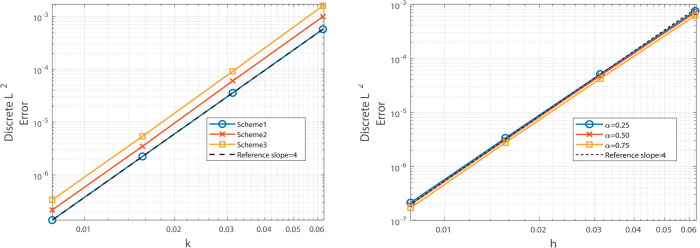
Illustrates the temporal convergence order (left) and spatial convergence order (right) of Besse relaxation compact difference scheme at α = 0.25, 0.50, and 0.75.

The graphical analysis of the numerical experimental results reveals that, for Example 2, the Besse relaxation difference scheme achieves a spatial convergence order of approximately two, as shown in Figs 5 and 6, while the Besse relaxation compact difference scheme attains a spatial convergence order of approximately four, as shown in Figs 7 and 8. Both schemes exhibit a temporal convergence order of approximately two. Compared to the results in Example 1, where the solution function exhibits singularity at *t* = 0, the smoothness of *u*_*tt*_(*x*,*t*) at *t* = 0 in Example 2 enables the numerical solution to achieve second-order accuracy in time. These findings are consistent with theoretical predictions, further validating the effectiveness of the proposed schemes for problems with smooth solutions.

**Example 3.** Assume the exact solution of (1)-(3) is


$$u(x,t)=t^{2}\;x^{\beta }(1-x{)}^{2},\;0<\beta <1.$$

The initial condition is u(x,0)=0, and the right–hand-side term is


$$f(x,t)=\frac{2\;t^{\;2-\alpha }}{\Gamma (3-\alpha )}\;x^{\beta }(1-x{)}^{2}-\;t^{2}[\beta (\beta -1)x^{\beta -2}-2\beta (\beta \;+\;1)x^{\beta -1}\;+\;(\beta \;+\;2)(\beta \;+\;1)x^{\beta }].$$

*Remark.* A *q-graded* mesh with *q* = 2 is adopted to resolve the endpoint singularity.

Tables 7 and 8 list the maximum errors and the associated convergence rates under different step sizes, with a fixed temporal grid *N* = 4096 and spatial grid *J* = 1024. One then refines either *h*_*x*_ or *h*_*t*_ to examine their respective influence on the error.

**Table 7:** Maximum errors and convergence orders with varying step sizes of the Besse Relaxation Difference Scheme — Example 3 (temporal grid number *N* = 4096 and spatial grid number *J* = 1024 fixed).

α	Spatial convergence order				Time convergence order			
	*J*	$E_{\infty }(h,\tau )$	${Rate}^{x}$	CPU(s)	*N*	$E_{\infty }(h,\tau )$	${Rate}^{t}$	CPU(s)
0.25	16	1.5527e-2	*	1.8653	32	7.0948e-2	*	2.1988
	32	3.8890e-3	2.0008	2.1348	64	3.2177e-2	1.2369	4.5523
	64	9.7130e-4	1.9977	5.4899	128	1.2689e-2	1.2482	9.3401
	128	2.4299e-4	2.0011	12.6142	256	5.3520e-3	1.2473	15.2630
0.50	16	1.4258e-2	*	1.9420	32	3.1431e-2	*	4.5877
	32	3.5667e-3	1.9877	7.4647	64	1.0979e-2	1.4796	6.0913
	64	8.8364e-4	1.9992	15.0089	128	4.0492e-3	1.5013	10.3982
	128	2.2351e-4	2.0038	18.1540	256	1.4091e-3	1.4885	17.6570
0.75	16	1.3267e-2	*	4.6689	32	1.2554e-2	*	3.1377
	32	3.3190e-3	1.9970	8.3673	64	3.8031e-3	1.7478	7.7504
	64	8.5852e-4	1.9823	13.9486	128	1.0989e-3	1.7488	12.2408
	128	2.1477e-4	2.0040	18.3447	256	3.3216e-4	1.7463	19.8331

**Table 8:** Maximum errors and convergence orders with varying step sizes of the Besse Relaxation Compact Difference Scheme — Example 3 (temporal grid number *N* = 4096 and spatial grid number *J* = 1024 fixed).

α	Spatial convergence order				Time convergence order			
	*J*	$E_{\infty }(h,\tau )$	${\text{Rate}}^{\text{x}}$	CPU(s)	*N*	$E_{\infty }(h,\tau )$	${\text{Rate}}^{\text{t}}$	CPU(s)
0.25	16	4.0380e-4	*	5.5746	32	6.9184e-3	*	7.0306
	32	2.5557e-5	3.9870	10.1318	64	2.7833e-3	1.2412	15.2235
	64	1.5988e-6	3.9903	24.2743	128	1.1268e-3	1.2447	20.7308
	128	1.0375e-7	3.9968	39.0964	256	4.5581e-4	1.2476	27.0644
0.50	16	7.4258e-4	*	6.6114	32	2.8733e-3	*	8.6402
	32	4.5667e-5	3.9879	13.1382	64	8.55383e-4	1.4877	14.0527
	64	2.9364e-6	3.9903	17.7261	128	3.2975e-4	1.4848	23.6404
	128	1.8430e-7	3.9960	24.4799	256	1.3174e-4	1.4950	35.1593
0.75	16	8.32675e-4	*	4.6591	32	1.1582e-3	*	14.7261
	32	5.2011e-5	4.0003	13.9501	64	3.3401e-4	1.7502	26.8627
	64	3.2507e-6	3.9929	29.7958	128	1.0869e-4	1.7445	33.8870
	128	2.0310e-7	3.9985	40.2632	256	3.3992e-5	1.7475	48.7473

From Table 7, one observes that the spatial discretization achieves *nearly second-order accuracy*: the *Rate* column approaches 2 as *J* doubles (i.e. *h*_*x*_ halves), and the errors $E_{s}(h_{x},\tau )$ decay proportionally to $h_{x}^{\;2}$. Turning to Table 8, the temporal convergence orders align well with theory, as the errors $E_{s}(h_{t})$ decrease steadily with finer time steps.

Overall, these numerical results confirm that the proposed method handles PDEs with unbounded derivatives effectively: the spatial scheme is nearly second-order accurate, and the temporal scheme exhibits convergence rates in close agreement with the predictions. The good consistency between expected and observed orders underscores the robustness and efficiency of the method for such challenging problems.

## 7 Conclusions

In this paper, we presented a fourth-order Besse relaxation compact difference scheme for solving nonlinear integro-differential equations. By combining Besse-type temporal relaxation with compact finite-difference spatial discretization, our method attains high spatial accuracy of $𝒪(h^{4})$ and robust temporal convergence, which adapts effectively to both smooth solutions and those displaying singularities at *t* = 0. Numerical experiments confirm these theoretical convergence rates, showcasing the capability of scheme to capture singular behavior with minimal loss of accuracy.

Beyond one-dimensional settings, the flexibility of the Besse relaxation framework allows straightforward extension to higher-dimensional domains. The compact difference approximation naturally generalizes to multi-dimensional stencils, preserving high-order accuracy in each spatial direction. In cases where the computational domain is non-rectangular or exhibits complex geometries, standard techniques such as coordinate transformations, unstructured meshes, or local stencil adaptations can be incorporated without compromising the core algorithmic structure. Hence, the Besse relaxation approach seamlessly interfaces with advanced grid-generation methods and tailored boundary conditions, maintaining its robust stability and convergence properties.

Taken together, these features indicate that our Besse relaxation compact scheme can be adapted to a wide class of fractional-order problems in higher dimensions. The method’s capacity to handle singular solutions, coupled with its high-order accuracy and computational efficiency, underscores its potential for diverse scientific and engineering applications. We anticipate that ongoing work involving multi-dimensional nonlinear integro-differential equations will further affirm the versatility and effectiveness of this approach.
