# Supplementary material for: Besse relaxation difference scheme for a nonlinear integro-differential equation
Source: PLoS One. 2025 Aug 29;20(8):e0327515. doi: 10.1371/journal.pone.0327515 (PMC12396723; doi:10.1371/journal.pone.0327515)
Supplement: Attachment — Submitted filename: Review_PONE-D-25-03186.pdf [file pone.0327515.s001.pdf]

In this paper the authors have provided Besse relaxation difference scheme for solving non linear integral differential equations. There are plenty of methods available for solving nonlinear integral differential equations. the authors did not provide a proper comparison based on the available methods and the advantage of the present method it was provided in the revised version of this article. in addition prover convergence analysis should be also provided. Hence this paper requires major revision.

There are a lot of mistakes in line 60. They wrote that G is continuously differentiable in a compact set and assuming its point wise bound as T goes to zero. If this is the case then how do you show the convergence of this improper integral or its validity.

The authors are mainly provided the numerical scheme with some little modification. But I see that this does not seem to be noble as neither the authors have provided the convergence analysis nor they are able to provide the stability of the present scheme For the system of differential equations ,partial differential equations and non linear problems with non local structures. this is very important to mention in the revised version, as they are already available in the works the authors must mention this works in the device version of this paper.

The authors must provide the convergence of the presence case in the revised version of this article.

The others must provide the stability estimate of the present scheme. Otherwise they can refer the avoid differences for the stability of the numerical scheme.

In the numerical example you have taken a function which is differentiable and its derivatives are bounded. However it will be more interesting to have a solution whose derivatives are unbounded as in that case the effectivity of the present method and the required smoothness will be clear. In general for singularly perturbed problems solution derivatives are unbounded from first order derivative onwards. they are all available.the authors must take at least two examples to show the convergence of the present methodology For those problems with derivatives are unbounded.

In page three and four the authors have derived the numerical scheme. however I am unable to see that required smoothness for this derivation. The stability of this scheme is also known at all clear. It must be addressed in the revised version. There are plenty of works availability on the stability as well as the convergence is obtained having minimum smoothness through a priori and aposteriori sense. They are available in- doi.org/10.1016/j.camwa.2023.04.004, https://doi.org/10.1111/sapm.12763, https://link.springer.com/article/10.1007/s11075-024-01918-5, DOI: 10.3934/nhm.2024048, https://onlinelibrary.wiley.com/doi/epdf/10.1002/fld.5294, https://doi.org/10.1016/j.apnum.2024.09.020. This works available in the above references. The authors must mention each and every references in the revised version of this work as the most of the difficult parts in the analysis are completely removed in the present version of this article. This work is of low value.

The graphs are not aligned properly. hence I am unable to understand the rate of acquisition of the present method through graphs. They are all added in the reverse.

I want to know what will be the expected time rate of accuracy based on the present method. Depending on the fractional derivative alpha? This must be explicitly mentioned with proper convergence analysis in the revised version of this article.

How do you extend the present methodology for nonlinear problems and for problems having non smooth data where you will not get enough smoothness for the convergence analysis. this must be mentioned in the revised version of this article.

There are several notations which are not defined. they are given an equation 4.

In addition I am unable to understand how come the equation arises after line 84 and equation 11.

The authors should provide the required compatibility conditions as without which one cannot guarantee the smoothness of the solution and hence the convergence of the present compact different scheme.

The wellposedness properties restricts conditions on the smoothenss and convergence analyss. However I am unable to see in a differential equation as an example where this existence theorem are used to prove. There are plenty of papers with the well posedness of the solution are proved by fixed point theory. They are available in https://doi.org/10.1016/j.camwa.2023.09.008, https://doi.org/10.1002/mma.9778, doi: 10.1007/s13398-023-01488-6, Journal of Computational and Applied Mathematics, 404, 113116, Elsevier, 2022, doi:10.1080/00207160.2019.1673892, http://doi.org/10.1002/mma.7369, doi:10.1002/cmm4.1047. the authors must mention each and every references in the revised version of this work as the present work is of low value.

In addition the authors have assumed that the given functions are not only smooth but also compatible. however this is nowhere mentioned. If the datas are not smooth and the problem is non compatible, the present methodology will not work. In these cases one can have boundary and interior layers phenomena where the solution derivatives are unbounded. The present work only considered the methodology based on bounded derivatives. this is too restrictive and does not have much applications in the real world . therefore much more complicated problems which is given in the papers- Journal of Difference Equations and Applications, 24, 3, 452-477, 2018, Journal of Computational and Applied Mathematics, 354, 533-544, Elsevier, 2019, Differential Equations and Dynamical Systems, 27(1-3):91-112, Springer, 2019, Journal of Computational and Applied Mathematics, 290, 16-25, 2015, Journal of Applied Mathematics and Computing, 41(1-2), 447-471, 2013, Australian Journal of Mathematical Analysis and Applications, 10(1), 1-17, 2013,doi:10.1142/S0219876212500521,. for system of differential equations as well as for non linear systems with incompatible as well as non smooth data must be mentioned in the revised version of this article.

What about the compatibility of the data first how much compatibility is assumed in the differential equation.
