# Supplementary material for: Besse relaxation difference scheme for a nonlinear integro-differential equation
Source: PLoS One. 2025 Aug 29;20(8):e0327515. doi: 10.1371/journal.pone.0327515 (PMC12396723; doi:10.1371/journal.pone.0327515)
Supplement: Attachment — Submitted filename: Response to reviewers.pdf [file pone.0327515.s002.pdf]

Response to Comments

We sincerely appreciate the invaluable feedback provided by the editor and all reviewers, which has greatly helped us enhance the quality of our manuscript. The reviewers' comments are presented in *italic font* below, with each query numbered for clarity. Our responses appear in regular font, and any revisions or additions to the manuscript are highlighted in red. We have carefully addressed each comment in detail, and we trust that these revisions adequately reflect your suggestions and improve the overall clarity and rigor of our work. Thank you once again for your thoughtful guidance and for the opportunity to refine our manuscript.

Reviewer #1.

Each and every comments must be addressed regarding convergence stability and example having unbdd derivative with recent modified works for complicated problems. This must be addressed in the revised version without which it cannot be processed further. I will recheck it.

Reply: We sincerely appreciate your insightful comments, which have significantly strengthened the rigor and clarity of our work. We have made substantial revisions to our manuscript based on your valuable feedback.

Firstly, we have added detailed proofs of stability and convergence for both the *Besse Relaxation Difference Scheme* and the *Besse Relaxation Compact Difference Scheme*, which now appear in Sections 5 and 6 of the revised manuscript (Pages 6–10, Lines 145–258). Meanwhile, we have briefly described this part of the work in the section of Abstract.

Secondly, We have expanded the Introduction section to include recent methods from the literature that address complex challenges in fractional partial differential equations, including a discussion of these methods' advantages and disadvantages, in order to clarify our motivation for using the Besse relaxation difference scheme (specifically, see Page 1, Lines 4–12, and Page 2, Lines 22–47).

Thirdly, we have added a new comparative experiment that adopts recently improved methods. Specifically, we have also conducted a comprehensive comparison between our proposed *Besse Relaxation Difference Scheme* and the two-term relaxation Crank–Nicolson method in terms of CPU time, revealing a 45% improvement at second order and a 35% improvement at fourth order (see Pages 13–15, Lines 297–307). The detailed comparison results are presented in Tables 1 and 2 below.

Table 1. CPU time comparison for different methods (temporal grid number $N = 4096$ and spatial grid number $J = 1024$ fixed)

α	Besse Relaxation difference Scheme				Crank–Nicolson scheme			
	J	$\mathrm{CPU}_x(s)$	N	$\mathrm{CPU}_t(s)$	J	$\mathrm{CPU}_x(s)$	N	$\mathrm{CPU}_t(s)$
0.25	16	1.8286	32	2.2123	16	3.3247	32	4.0224
	32	2.1051	64	4.5729	32	3.8275	64	8.3144
	64	5.5631	128	9.4028	64	10.1147	128	17.0960
	128	12.7636	256	18.2695	128	23.2065	256	33.2173
0.50	16	1.9036	32	2.4839	16	3.4611	32	4.5162
	32	1.9314	64	4.3301	32	3.5116	64	7.8729
	64	5.5885	128	9.1678	64	10.1609	128	16.6687
	128	13.0534	256	18.2089	128	23.7335	256	33.1071
0.75	16	1.5953	32	2.2142	16	2.9005	32	4.0258
	32	2.3458	64	4.3942	32	4.2651	64	7.9894
	64	5.6509	128	8.9153	64	11.2744	128	16.2996
	128	13.4627	256	18.1666	128	25.4776	256	32.9902

Table 2. CPU time comparison for different methods (temporal grid number $N = 4096$ and spatial grid number $J = 1024$ fixed)

α	Besse Relaxation difference Scheme				Crank–Nicolson scheme			
	J	$\mathrm{CPU}_x(s)$	N	$\mathrm{CPU}_t(s)$	J	$\mathrm{CPU}_x(s)$	N	$\mathrm{CPU}_t(s)$
0.25	16	10.5005	32	2.4483	16	16.0546	32	3.8666
	32	12.2225	64	5.6630	32	17.9946	64	8.7123
	64	22.8864	128	15.2265	64	34.2098	128	23.4254
	128	63.6645	256	17.3352	128	98.9454	256	23.6695
0.50	16	10.5968	32	2.4839	16	16.3028	32	3.8214
	32	11.2566	64	4.3301	32	17.3186	64	6.6617
	64	24.8879	128	9.1678	64	37.2889	128	15.0243
	128	36.0921	256	18.2089	128	56.5267	256	27.6695
0.75	16	15.3389	32	13.2507	16	24.5980	32	21.3852
	32	25.5537	64	38.4733	32	37.3134	64	54.9897
	64	65.1153	128	64.3375	64	98.8774	128	99.9808
	128	98.1240	256	78.5537	128	148.9860	256	123.1518

Finally, we have added a novel numerical example focusing on unbounded derivatives, presented in Section 6 (see Pages 15–16, Lines 318–330). These additions effectively validate the applicability of our method to real-world fractional order problems. Similarly, we have briefly described this part of the work in the section of Abstract.

Reviewer #2.

The authors present a Besse type finite difference relaxation scheme for approximating solutions of a nonlinear integer-differential equation modelling systems with memory and nonlocal effects. Some comments are in order.

Reviewer Comment 2.1 — *p2. Line 22, "... Georgios and Zoularis... ",Is that 2 different authors? From [11] it seems it is just one author.*

Reply: Thank you for pointing out the issue regarding "Georgios and Zoularis". In accordance with your suggestion, we have corrected "Georgios and Zoularis" to "Zouraris" and highlighted this amendment in red in the fourth paragraph of the Introduction. Meanwhile, we have thoroughly reviewed the entire manuscript to avoid the recurrence of similar issues.

Reviewer Comment 2.2 — *p2, lines 30–31, the context and wording is not clear.*

Reply: Thank you for pointing out that the wording in lines 30–31 was unclear. Based on your suggestion, we have revised this sentence as follows:
Before: Although Besse relaxation difference schemes can improve computational efficiency using the simplification of nonlinear term handling, they are explicit methods and usually perform weak accuracy and stability in complex boundary conditions or nonlinear problems.
Changed: However, given that the second-order Besse relaxation difference scheme is still limited to second-order spatial accuracy, there is an urgent need for compact variants with higher algebraic complexity to further improve computational accuracy when solving complex partial differential equations [16].

Reviewer Comment 2.3 — *In the introduction the authors should clarify, maybe in a separate paragraph, what are they novelties introduced in this paper, and how their approach compares to the other methods in the literature for this problem, in terms of accuracy and computational cost.*

Reply: We greatly appreciate this valuable suggestion. In response, we have revised the section of Introduction. Specifically, a detailed explanation of the innovative aspects of our study has been revised and presented on Pages 2, Lines 48–63. In short, our contributions are as follows:

(1) Two novel time–space discretization schemes, namely the Besse relaxation and Besse relaxation compact schemes, are proposed to enhance stability and improve accuracy.

(2) Rigorous stability and convergence analyses confirm that the schemes are unconditionally stable and convergent.

(3) Numerical experiments on equations with singular solutions, smooth solutions, and unbounded derivatives demonstrate the robustness and adaptability of our approach.

In addition, we have further clarified how our approach compares to existing methods in the literature with respect to accuracy and computational cost (see Pages 2, Lines 22–47 for more details).

Reviewer Comment 2.4 — *p7, line 138, In Example 1 what are the sizes of the space and time intervals? The same question for Example 2 in p.9, line 163.*

Reply: Thank you for asking about the sizes of the space and time intervals in Examples 1, 2. In both examples, we have chosen a temporal grid number $N = 4096$ and a spatial grid number $J = 1024$. Specifically, the time interval is $[0, T]$, with $\Delta t = T/N$, and the spatial domain is $[x_{\min}, x_{\max}]$, with $\Delta x = (x_{\max} - x_{\min})/J$.Meanwhile, following the suggestions from other reviewers, we have added Example 3. In Example 3, we likewise use the same temporal and spatial grid settings. In addition, we have clarified these details in the revised manuscript (see Pages 11, Lines 275–276).

Reviewer Comment 2.5 — *The errors and rates are reported using the maximum norm. It would be beneficial if they are also reported in the discrete L^2 norm.*

Reply: Thank you for your valuable suggestion. In accordance with your request, we have included an error analysis under the discrete L^2 norm for Examples 1 and 2 to enhance the credibility of our results (see Pages 11–15, Lines 277–317 for more details). The partial detailed comparison results are presented in Tables 3 and 4 below. Clearly, our proposed method can still achieve the expected order of convergence under the discrete L^2 norm.

Table 3. Maximum errors and convergence orders with varying step sizes (temporal grid number $N = 4096$ and spatial grid number $J = 1024$ fixed)

α	spatial convergence order				time convergence order			
	J	$E_2(h,\tau)$	$\mathrm{Rate}_x^{(2)}$	CPU(s)	N	$E_2(h,\tau)$	$\mathrm{Rate}_t^{(2)}$	CPU(s)
0.25	16	1.2247e-2	*	3.0049	32	6.2873e-2	*	4.6987
	32	3.1057e-3	1.9742	5.0013	64	2.5167e-2	1.2423	8.9855
	64	7.8621e-4	1.9820	8.2513	128	1.0708e-2	1.2485	14.4606
	128	1.9813e-4	1.9890	9.5106	256	4.3580e-3	1.2493	33.5423
0.50	16	1.1123e-2	*	1.8945	32	2.7207e-2	*	4.2862
	32	2.7820e-3	1.9964	4.2312	64	9.2458e-3	1.4968	9.1254
	64	6.9631e-4	1.9983	7.7035	128	3.2683e-3	1.4979	18.5481
	128	1.7433e-4	1.9981	9.5414	256	1.1544e-3	1.4990	37.3611
0.75	16	1.0458e-2	*	1.1565	32	1.0996e-2	*	3.4515
	32	2.5940e-3	1.9796	2.5688	64	2.2198e-3	1.7458	10.0195
	64	6.5491e-4	1.9835	4.7173	128	9.4925e-4	1.7473	16.4823
	128	1.6375e-4	1.9849	9.4720	256	2.9172e-4	1.7495	29.3727

Table 4. Maximum errors and convergence orders with varying step sizes (temporal grid number $N = 4096$ and spatial grid number $J = 1024$ fixed)

α	spatial convergence order				time convergence order			
	J	$E_2(h,\tau)$	$\mathrm{Rate}_x^{(2)}$	CPU(s)	N	$E_2(h,\tau)$	$\mathrm{Rate}_t^{(2)}$	CPU(s)
0.25	16	9.7789e-4	*	15.2859	32	1.3598e-2	*	6.6382
	32	6.2011e-5	3.9824	27.7653	64	5.7173e-3	1.2458	14.7034
	64	3.9079e-6	3.9907	31.6538	128	2.3790e-3	1.2466	20.3718
	128	2.4088e-7	3.9982	45.9277	256	1.0250e-3	1.2483	22.5638
0.50	16	8.0418e-4	*	21.1878	32	2.0258e-3	*	3.7287
	32	5.1028e-5	3.9714	19.7357	64	7.5510e-4	1.4945	9.3906
	64	3.2037e-6	3.9944	56.5537	128	2.8859e-4	1.4976	17.2667
	128	2.0013e-7	3.9980	24.4799	256	1.0183e-4	1.4991	25.4824
0.75	16	7.0347e-4	*	41.1666	32	7.7354e-4	*	13.7724
	32	4.5076e-5	3.9594	50.4728	64	2.0770e-4	1.7455	29.0614
	64	2.8061e-6	3.9642	66.6870	128	6.9821e-5	1.7470	41.0010
	128	1.7529e-7	3.9826	105.3017	256	2.0632e-5	1.7489	70.5891

Reviewer Comment 2.6 — *The authors, in Tables 1 and 2 reported the required CPU time. How does this time compares, if a non-relaxation method is used to approximate solutions of model (1)? A comparison table would very beneficial.*

Reply: Thank you very much for your valuable feedback. In accordance with your recommendation, we applied the non-relaxation Crank – Nicolson method to solve Example 1. The results indicate that, compared with non-relaxation method, our proposed approach achieves approximately a 45% reduction in CPU time at second-order accuracy and about a 35% reduction at fourth-order accuracy, as detailed in the comparison presented on Pages 13–15 ,Lines 297–307. The detailed comparison results are presented in Tables 5 and 6 below.

Table 5. CPU time comparison for different methods (temporal grid number $N = 4096$ and spatial grid number $J = 1024$ fixed)

α	Besse Relaxation difference Scheme				Crank–Nicolson scheme			
	J	$\mathrm{CPU}_x(s)$	N	$\mathrm{CPU}_t(s)$	J	$\mathrm{CPU}_x(s)$	N	$\mathrm{CPU}_t(s)$
0.25	16	1.8286	32	2.2123	16	3.3247	32	4.0224
	32	2.1051	64	4.5729	32	3.8275	64	8.3144
	64	5.5631	128	9.4028	64	10.1147	128	17.0960
	128	12.7636	256	18.2695	128	23.2065	256	33.2173
0.50	16	1.9036	32	2.4839	16	3.4611	32	4.5162
	32	1.9314	64	4.3301	32	3.5116	64	7.8729
	64	5.5885	128	9.1678	64	10.1609	128	16.6687
	128	13.0534	256	18.2089	128	23.7335	256	33.1071
0.75	16	1.5953	32	2.2142	16	2.9005	32	4.0258
	32	2.3458	64	4.3942	32	4.2651	64	7.9894
	64	5.6509	128	8.9153	64	11.2744	128	16.2996
	128	13.4627	256	18.1666	128	25.4776	256	32.9902

Table 6. CPU time comparison for different methods (temporal grid number $N = 4096$ and spatial grid number $J = 1024$ fixed)

α	Besse Relaxation difference Scheme				Crank–Nicolson scheme			
	J	$\mathrm{CPU}_x(s)$	N	$\mathrm{CPU}_t(s)$	J	$\mathrm{CPU}_x(s)$	N	$\mathrm{CPU}_t(s)$
0.25	16	10.5005	32	2.4483	16	16.0546	32	3.8666
	32	12.2225	64	5.6630	32	17.9946	64	8.7123
	64	22.8864	128	15.2265	64	34.2098	128	23.4254
	128	63.6645	256	17.3352	128	98.9454	256	23.6695
0.50	16	10.5968	32	2.4839	16	16.3028	32	3.8214
	32	11.2566	64	4.3301	32	17.3186	64	6.6617
	64	24.8879	128	9.1678	64	37.2889	128	15.0243
	128	36.0921	256	18.2089	128	56.5267	256	27.6695
0.75	16	15.3389	32	13.2507	16	24.5980	32	21.3852
	32	25.5537	64	38.4733	32	37.3134	64	54.9897
	64	65.1153	128	64.3375	64	98.8774	128	99.9808
	128	98.1240	256	78.5537	128	148.9860	256	123.1518

Reviewer Comment 2.7 — *Can the authors comment on the extension and applicability of their approach in 2-space dimensions with non rectangular domains?*

Reply: We sincerely appreciate the reviewer's interest in extending our fourth-order Besse relaxation compact difference scheme to two-dimensional settings and non-rectangular domains. Based on your feedback, we have revised the section of Conclusion. The detailed modifications can be found on Pages 17, Lines 338–351. In short, the potential for extending and applying our method in two-dimensional space and non-rectangular domains can be summarized as follows:

(1) In terms of adaptability, the compact stencil naturally applies in each spatial direction while preserving high-order accuracy, and the temporal Besse relaxation remains effective for multi-dimensional fractional systems in 2-space dimensions. Meanwhile, for more complex geometries, we can employ boundary-fitted coordinates or unstructured meshes, incorporating polynomial reconstructions or ghost point methods to maintain stability and convergence.

(2) In terms of generalization, we plan to investigate a wider range of mesh topologies, validate our approach on benchmark problems, and refine implementation details (including parallelization) for large-scale simulations. We trust that this explanation clarifies our method's adaptability and thank the reviewer for prompting us to elaborate.
